# Die tibiale Derotationsosteotomie bei Kniegelenkspathologien: Indikationen, Operationstechnik und Übersicht über die aktuelle Literatur

**DOI:** 10.1007/s00132-025-04675-y

**Published:** 2025-07-14

**Authors:** Michael Liebensteiner, Clelia Appel-Ersek, Gerhard Kaufmann, Markus Neubauer, Johannes Neugebauer, Dietmar Dammerer

**Affiliations:** 1 Orthopädie f. Hüfte, Knie & Fuß im Zentrum, Innrain 2, Innsbruck, Österreich; 2 Privatklinik Kettenbrücke, Sennstr. 1, Innsbruck, Österreich; 3 A.ö. Krankenhaus St. Vinzenz Zams, Sanatoriumstr. 43, Zams, Österreich; 4 Universitätsklinikum Krems, Mitterweg 10, 3500 Krems, Österreich; 5 Karl Landsteiner Universität für Gesundheitswissenschaften, Dr. Karl-Dorrek-Straße 30, 3500 Krems, Österreich

**Keywords:** Femur, Patellofemoralgelenk, Patient reported outcome, Biomechanik, Torsionsanomalie, Femur, Patellofemoral joint, Patient reported outcome, Biomechanics, Torsion abnormality

## Abstract

Pathologische Torsionen des Oberschenkels und Unterschenkels sind häufig. Die negativen Auswirkungen auf das Knie sind evident und betreffen nicht nur das Patellofemoralgelenk. Die Verdachtsdiagnose auf eine Maltorsion wird durch die physische Untersuchung generiert, und durch die Schichtbildgebung verifiziert (MRT oder CT). Das von den Autoren durchgeführte Literatur-Review zum Thema Derotationsosteotomien an der Tibia erbrachte 35 Studien (1562 Osteotomien). Fast alle Studien berichteten, auf Basis der „patient-reported outcomes“ (Kujala, SF-36, Lysholm, KOOS, VAS und generelle Patientenzufriedenheit), von einer deutlichen Verbesserung im Vergleich zu vor der Operation. Aufgrund des Fehlens von vergleichenden Studien ist bis dato die beste Operationstechnik für die tibiale Derotationsosteotomie noch unbestimmt. Für komplexe Fälle, bei welchen Deformitäten in mehreren Körperebenen korrigiert werden sollen (z. B. Maltorsion und Valgus-Varus-Deformität), scheint die dreidimensionale Planung und Umsetzung mittels patientenspezifischer Schnittblöcke vielversprechend.

## Abstract (en)

Pathological torsions of the femur and tibia are common. The negative effects on the knee are evident and do not only affect the patellofemoral joint. The suspected diagnosis of maltorsion is generated by physical examination and verified by tomographic imaging (MRI or CT). The literature review regarding tibial derotation osteotomies conducted by the authors yielded 35 studies (1562 osteotomies). Almost all of the studies reported a significant improvement compared to preoperatively based on patient-reported outcomes (Kujala, SF-36, Lysholm, KOOS, VAS and general patient wellbeing). Due to the lack of comparative studies, the best surgical technique for tibial derotation osteotomy is still undetermined. For complex cases in which deformities in several body planes are to be corrected (e.g., maltorsion and valgus varus deformity), three-dimensional planning and implementation using patient-specific incision blocks seems promising.

## Symptome

Die Rolle der pathologischen Torsion der Tibia in der Entstehung von Kniegelenksbeschwerden ist gut dokumentiert [7, 38]. Die Autoren nennen als Symptome a) patellofemorale Schmerzen bzw. Instabilität, b) Stolpern, verursacht durch „toeing-in“ bzw. „toeing-out“ und c) ästhetischen Leidensdruck. Im Falle einer exzessiven Außentorsion des Unterschenkels kommt es während der Bewegung des Kniegelenks beim Springen und Laufen zu einem übermäßigen Valgusstress (externes Valgusmoment), meist bedingt durch eine kompensatorische Innenrotation des Femurs [2, 7, 12]. Daher scheint es plausibel, dass am Kniegelenk nicht immer nur patellofemorale Beschwerden resultieren, sondern durchaus auch tibiofemorale.

## Physische Untersuchung

**Abb. 1:**
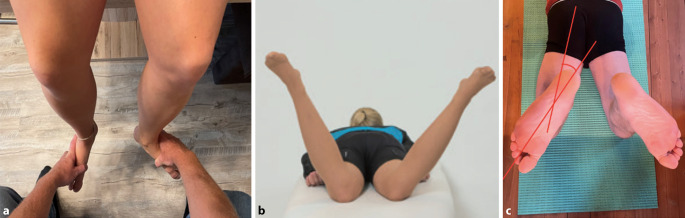
Klinische Untersuchung von „foot progression angle“ (**a**), Hüftrotation (**b**) und „thigh foot angle“ (**c**)

In der Untersuchung in Stand und Gang wird auf eine Beinlängendifferenz, auf den „foot progression angle“ (FPA, Winkel des Fußes in Relation zur Gehrichtung) auf Deformitäten in der frontalen Ebene und ein Kniestreckdefizit bzw. Genu recurvatum geachtet. Ebenso wird das Rückfußalignment und die Patellaposition beachtet (Patellastrabismus o. ä.). Im Sitzen auf der Liege sollten die Grenzen der Knierotation im Seitenvergleich untersucht werden. In Bauchlage testet man die Grenzen der Hüftrotation und den „thigh foot angle“ (TFA) (Abb. 1).

**Abb. 2:**
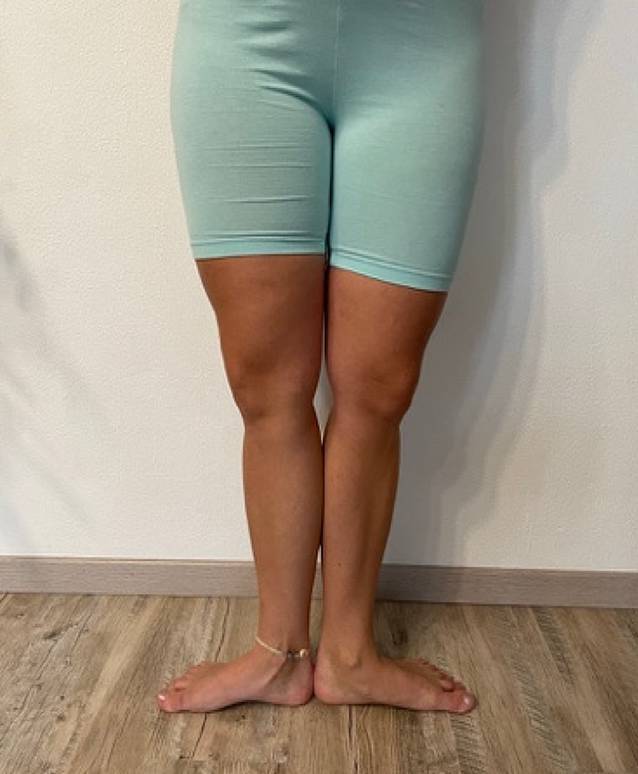
Exzessive Außenrotation der gesamten unteren Extremität

In vielen Fällen gelangt man durch die physische Untersuchung lediglich zu einem Verdacht. Die präzise Verortung der Maltorsion bleibt der Bildgebung überlassen. Im Beispiel der Abb. 2 fällt eine exzessive Außenrotationsfähigkeit der gesamten unteren Extremität auf.

Als Ursachen kommen infrage (einzeln und kombiniert): Retroversion des Azetabulums, exzessive Beweglichkeit im Hüftgelenk z. B. durch Hyperlaxizität/Kollagenerkrankung, Maltorsion des Oberschenkels, Exzessive Beweglichkeit im Kniegelenk z. B. durch Hyperlaxizität (Knieversion), Maltorsion des Unterschenkels, Hyperlaxizität im Rückfuß. Anhand dieses Beispiels wird deutlich, dass es in den meisten Fällen einer Schichtbildgebung zur genaueren Analyse bedarf.

## Bildgebung und Operationsindikation

Ähnlich der Analyse der Torsion am Oberschenkel gibt es auch bei der Analyse der Torsion am Unterschenkel anhand einer Computertomographie (CT) eine Unzahl von beschriebenen Methoden. Einen Auszug aus jenen Methoden liefert folgende Tab. 1.

**Tab. 1:** Vermessungstechniken der tibialen Torsion

Quelle	Methode der Vermessung (graphisch)
Waidelich, Strecker, Schneider. Rofo 1992 Sep;157(3) [45]	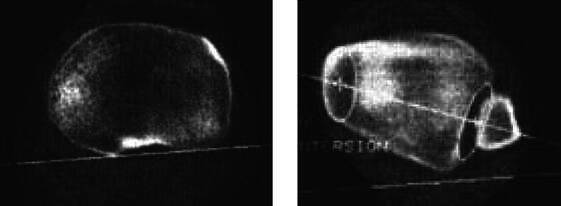
Jakob, Haertel, Stussi E Bone Joint Surg Br. 1980 62-B(2):238-242 [21]	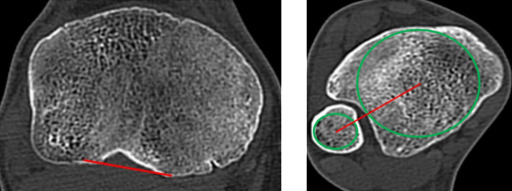
Goutallier et al. J Bone Joint Surg Am 2006 Nov;88(11) [18]	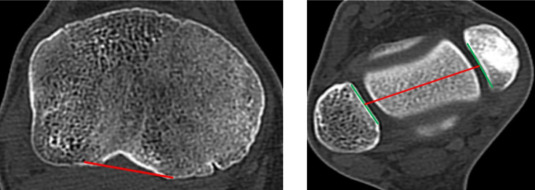
Stephen et al. Am J Sports Med 2021 Mar;49(3) [41]	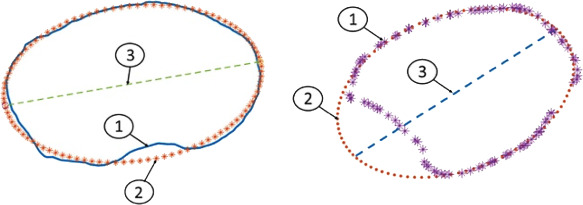
Hoch et al. Insights Imaging 2021 Feb 15;12(1) [19]	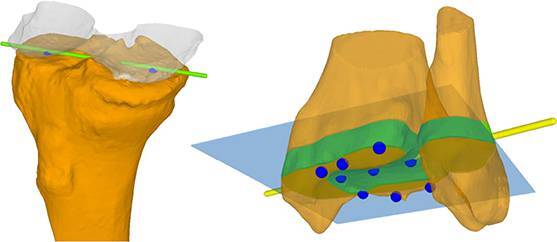
Schock et al. Sci Rep 2021 Dec 1;11 (1) [36]	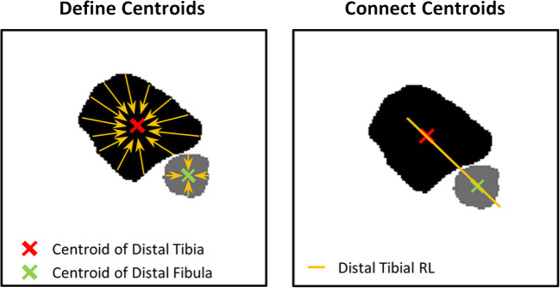

Je nach angewendeter Messmethode resultieren leicht variable Ergebnisse. Die Thematik wird zusätzlich erschwert durch das Fehlen eines eindeutigen radiologischen Grenzwertes für eine Operationsindikation (exzessive Außentorsion als auch exzessive Innentorsion). Letztere muss fast immer in Zusammenschau mit anderen Aspekten überlegt werden (FPA, gleichzeitige Korrektur am Oberschenkel etc.).

In Hinblick auf die Grenzen der Normalität der Torsion am Unterschenkel werden von den Autoren folgende Überlegungen angestellt. Mehrere Studien untersuchten die durchschnittliche Torsion am Unterschenkel von Gesunden [15, 28, 33]. Alle drei Publikation berichteten über Kollektive zwischen 100 und 200 Personen und konstatieren je nach Studien durchschnittliche Außentorsionswerte von 31° [33] und 29°[15] und 31° bzw. 34° (2 Messmethoden) [28]. Eine weitere Studie untersuchte die durchschnittlichen Torsionswerte an der Angio-CT von Gefäßpatienten und berichtete von einem Durchschnitt von 26° Außentorsion [44]. Auf Basis dieser 4 Studien kann von einem durchschnittlichen Normalwert von ca. 30° Außentorsion ausgegangen werden.

In den Händen der Autoren findet eine innenrotierende Korrekturosteotomie statt im Falle von Außentorsion von > 40° und gleichzeitigen Symptomen wie oben genannt. Die Indikation zur einer außendrehenden Korrekturosteotomie (meist neuroorthopädische Fälle) ist bei einer Außentorsion von < 15° und gleichzeitigem deutlichem „toeing-in“ (Stolpern) zu erwägen.

Die Vermessung erfolgt aus strahlenhygienischen Gründen meist mittels Becken-Bein-MRT.

## Lokalisation und Technik der Osteotomie

### Osteotomie proximal der Tuberositas tibiae

Eine Reihe von Autoren publizierte ihre Ergebnisse der derotierenden Tibiaosteotomie oberhalb der Tuberositas tibiae [6, 8, 22, 27, 31, 40]. Als Vorteile dürfen die große knöcherne Konsolidierungsfläche gelten als auch der Umstand, dass bei korrigierender Innenrotation die Tuberositas im gleichen Zuge medialisiert wird. Nachteilig ist bei dieser Technik, dass oberhalb der Osteotomie nur relativ wenig Platz für die Fixation verbleibt.

### Osteotomie auf Höhe der Tuberositas tibiae mit aszendierendem Schenkel

Die Technik der Osteotomie mit aszendierendem Schenkel (biplanar) direkt hinter der Tuberositas tibiae kompensiert die Nachteile der vorgenannten Methode. Bei immer noch großzügiger knöcherner Konsolidierungsfläche besteht nun ausreichend Platz auch für 2 Schraubenreihen des Osteosynthesematerials. Auch bei dieser Methode kommt es bei Innendrehung des distalen Segmentes zu einer automatischen Medialisierung der Tuberositas tibiae. Auch für diese Technik existieren bereits mehrere Publikationen [9, 10, 25].

### Osteotomie auf Höhe der Tuberositas tibiae mit zusätzlicher Tuberositasosteotomie

Die Technik der Osteotomie auf Höhe der Tuberositas tibiae mit gleichzeitiger Ablösung selbiger wurde von drei Autorengruppen berichtet [6, 11, 16]. Es besteht an dieser Stelle eine immer noch günstige metaphysäre Knochenheilung, ausreichend Platz proximal für die Fixation des Osteosynthesematerials und die Platzierung der Tuberositas ist am Abschluss der Operation theoretisch frei wählbar.

### Osteotomie distal der Tuberositas tibiae (meta-diaphysärer Übergang)

Diese Technik wurde bisher nur von wenigen Autoren gewählt [24, 46]. Anzunehmen ist ein höheres Risiko für eine Pseudarthrose aufgrund der kleineren Osteotomiefläche bei gleichzeitig monoplanarer Osteotomietechnik. Im Gegensatz zu den zuvor genannten Methoden kommt es zu keinem automatischen Mitdrehen der Tuberositas tibiae (keine gleichzeitige Medialisierung).

### Osteotomie diaphysär

Diese Technik wird in der Literatur lediglich von 2 Publikationen gestützt, diese aus ein und derselben Autorengruppe [42, 43]. Durch die Lokalisation am Schaft bietet sich ein Marknagel für die Osteosynthese an. Mit einem etwas erhöhten Risiko für ein Kompartmentsyndrom muss gerechnet werden.

### Supramalleoläre Osteotomie

Diese Variante kommt in erster Linie im Feld der Kinder- und Neuroorthopädie zum Einsatz und wird von vielen Autoren sowohl für die innen- als auch außenrotierende Korrektur beschrieben [2, 3, 13, 23, 26, 32, 34, 35, 39].

Siehe Abb. 3.

**Abb. 3:**
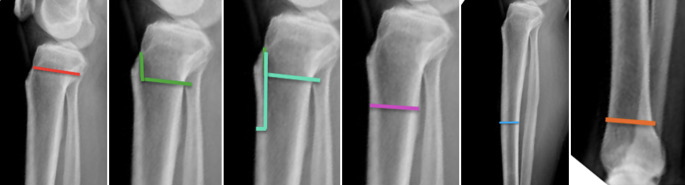
Schematische Darstellung der unterschiedlichen Osteotomielokalisationen

Ebenso wie die Vielzahl an möglichen Etagen für die Osteotomie sind auch einige weitere chirurgische Details noch ohne Konsensus. Unter anderem ist unklar, ab welchem Ausmaß der rotatorischen Korrektur eine zusätzliche Fibulaosteotomie empfehlenswert ist. Ähnlich ungewiss ist, welche *maximale* Korrektur prinzipiell erfolgen darf und in welchen Fällen ein zusätzliches „Release/Dekompression“ des Nervus peroneus erfolgen sollte. Selbiges gilt auch für etwaige prophylaktische Fasziotomien des anterioren Unterschenkelkompartments [10, 38, 46].

## Übersicht über die aktuelle Literatur

Die Literatursuche erfolgte mit dem Suchbegriff „(torsion OR anteversion) AND ([lower AND leg] OR tibia) AND osteotomy“ und folgenden Filtereinstellungen: a) nur deutsche oder englische Sprache, b) keine Case-Reports, c) nur klinische Originalarbeiten d. h. keine Kadaverstudien, keine Tierstudien, keine biomechanischen Simulationen. Die Suche ergab 369 Treffer, welche weiter auf Basis des Abstracts untersucht wurden. Daraus ergaben sich 35 Publikationen welche als relevant betrachtet wurden und auf Volltextebene analysiert wurden [1–14, 16, 17, 20, 22–27, 29–32, 34, 35, 37, 39, 40, 42, 43, 46].

Die genannten 35 Studien berichteten von zusammengefasst 1562 derotierenden Osteotomien der Tibia bzw. des Unterschenkels (überwiegend Evidenzlevel 4). 10 Studien thematisierten ausschließlich Patienten mit neuroorthopädischen Erkrankungen welche mit einer Maltorsion einher gingen (Zerebralparese, Myelomeningozele etc.). 18 weitere Arbeiten berichteten von Fällen ohne neurologische Pathologie (13 mit eindeutiger patellofemoraler Symptomatik, 5 mit genereller Gonalgie und eingeschränkter Gehfunktion). 6 Studien beinhalteten einen sog. Fallmix und eine Studie konnte zu dem Krankheitsbild keine näheren Angaben liefern. Bezüglich der Lokalisation der Osteotomie gliederten sich die 35 Studien wie in Tab. 2 aufgelistet.

**Tab. 2:** Übersicht über die unterschiedlichen Lokalisationen der Osteotomie in den analysierten Studien

Lokalisation der Osteotomie	Anzahl der Studien
Osteotomie proximal der Tuberositas tibiae	5
Osteotomie auf Höhe der Tuberositas tibiae mit aszendierendem Schenkel	3
Osteotomie auf Höhe der Tuberositas tibiae mit zusätzlicher Tuberositasosteotomie	2
Osteotomie distal der Tuberositas tibiae (meta-diaphysärer Übergang)	2
Osteotomie diaphysär	2
Osteotomie supramalleolär	16
Mix	5

Jene 18 Studien (537 Osteotomien) welche von Patienten mit Maltorsion ohne neurologische Erkrankung berichten sollen an dieser Stelle detaillierter analysiert werden [2, 6–11, 16, 22, 23, 25–27, 31, 35, 40, 42, 43]. 15 der 18 Studien beinhalten ausschließlich Fälle mit derotierender Korrektur nach innen bei präoperativ exzessiver Außentorsion. Bei 3 der 18 Studien handelt es sich um einen Fallmix, beinhalten also Fälle mit derotierender Korrektur nach innen und Fälle mit derotierender Korrektur nach außen. In 5 der 18 Arbeiten wurden Patienten miteingeschlossen, welche in gleicher Sitzung auch eine derotierende Osteotomie am Femur erhielten. Das Alter der Patienten war über die 18 Studien gemittelt 20,7 Jahre. Die Osteosynthese erfolgte je nach Studie und Fall mit einer Vielzahl von Implantaten (Marknägel, Platten, Klammer, Fixateur extern). Die 18 Studien bedienten sich unterschiedlicher Outcome-Parameter. Hinsichtlich sog. „patient-reported outcomes“ kamen Kujala, SF-36, Lysholm, KOOS, VAS und generelle Patientenzufriedenheit zum Einsatz. Komplikationsraten, radiologisches Ergebnis und Ganganalyse wurden von manchen Autoren ebenfalls verwendet. Fast alle der 18 Studien berichteten, auf Basis der o. g. „patient-reported outcomes“, von einer deutlichen Verbesserung im Vergleich zu vor der Operation. Die Komplikationsrate spannte sich, je nach Studie, zwischen 0 und 13 % aus, am häufigsten wurden Pseudarthrosen, Peroneusparesen sowie Irritationen durch das Osteosynthesematerial beschrieben.

Von o. g. 18 Studien sollen an dieser Stelle noch 3 Studien genau diskutiert werden, da diese aus den anderen hervorstechen [11, 27, 31]. Die Studie von Manilov et al. stellt die Arbeit mit der größten Fallzahl dar [27]. Die Autoren schlossen 60 Patienten ein, welche an patellofemoralen Schmerzen litten in Zusammenhang mit einer Maltorsion des Unterschenkels. Die Autoren behandelten die Patienten mit einer derotierenden Osteotomie oberhalb der Tuberositas tibiae und berichteten von signifikanten Verbesserungen in den Parametern Kujala Score und Fulkerson Score innerhalb 66 Monaten Nachuntersuchungszeitraum. Drexler et al. [11] publizierten die Studie mit dem längsten Nachuntersuchungszeitraum innerhalb o. g. Arbeiten, mit einem medianen Follow-up von 84 Monaten. Untersucht wurden Patienten mit lateraler Patelladislokation bei Maltorsion, welche mit derotierender Osteotomie auf Höhe der Tuberositas (unter gleichzeitiger Ablösung der Tuberositas) behandelt wurden. Auf Basis der angewendeten Fragebögen (KSS, Kujala, WOMAC, SF-12, VAS) berichteten die Autoren von signifikanten Verbesserungen. Die Studie von Paulos et al. [31] verdient besondere Aufmerksamkeit aufgrund des Vergleichs von Patienten mit Patellainstabilität behandelt mit supratuberositärer Derotation vs. Patienten mit lediglich Medialisierung der Tuberositas. Anhand der Nachuntersuchung mittels SF-36, KOOS, Kujala und Ganganalyse konstatieren die Autoren beiden Gruppen eine signifikante Verbesserung über die Zeit (Längsschnitt). Allerdings wurde ebenfalls festgestellt, dass es in der Derotationsgruppe zu signifikant besseren Ergebnissen kam als in der Gruppe mit lediglich Tuberositastransfer.

## Fallbeispiele

### Fall 1

**Abb. 4:**
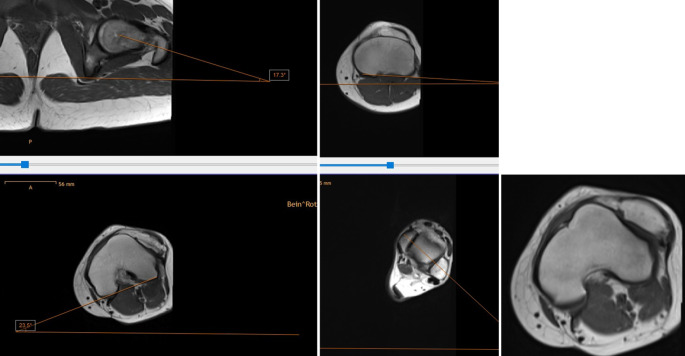
Fall 1: Präoperative Bildgebung mittels Becken-Bein-MRT, Vermessung der femoralen Torsion (*links*), sowie der tibialen Torsion (*rechts*) bei permanent luxierter Patella (*rechts unten*)

20-jährige Patientin mit permanenter lateraler Patelladislokation bei zugrundeliegender Trochleadysplasie und einer Oberschenkeltorsion von 40° und einer Unterschenkeltorsion von 40° und pathologischen Werten für die Parameter TT-PCL und TT-TG (Abb. 4).

Der Behandlungsplan war wie folgt:

1. Derotierende distale Femurosteotomie
2. Derotierende proximale Tibiaosteotomie mit gleichzeitiger Medialisierung der Tuberositas (biplanare Osteotomie)
3. Trochleaplastik
4. MPFL-Rekonstruktion
5. evtl. Quadrizepsverlängerung

**Abb. 5:**
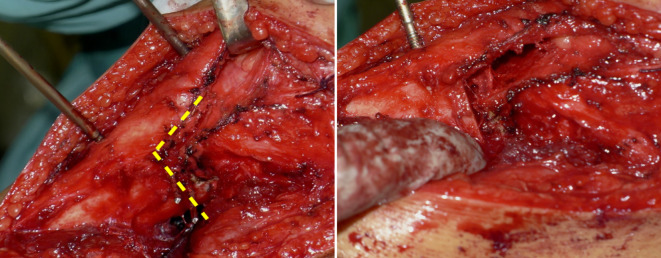
Fall 1: Intraoperativer Situs, *gelbe Linie* markiert die Lokalisation der biplanaren Osteotomie an der Tibia

**Abb. 6:**
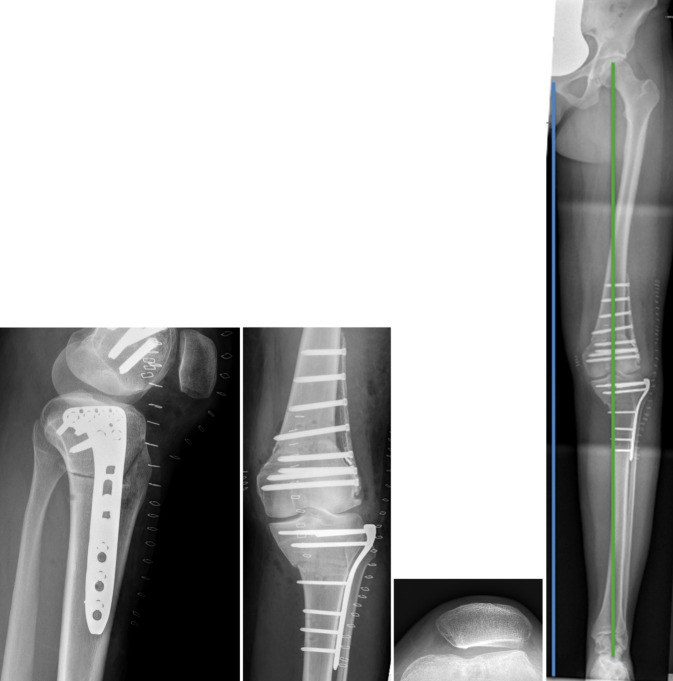
Fall 1: Postoperatives Ergebnis, Röntgenbilder nach femoraler und tibialer Derotation, die Patella liegt wieder zentral in der Trochlea und die Beinachse wurde korrigiert

Intraoperative Umsetzung wie geplant (Abb. 5), jedoch keine Quadrizepsverlängerung notwendig. Patientin mit dem Ergebnis sehr zufrieden (Abb. 6).

### Fall 2

**Abb. 7:**
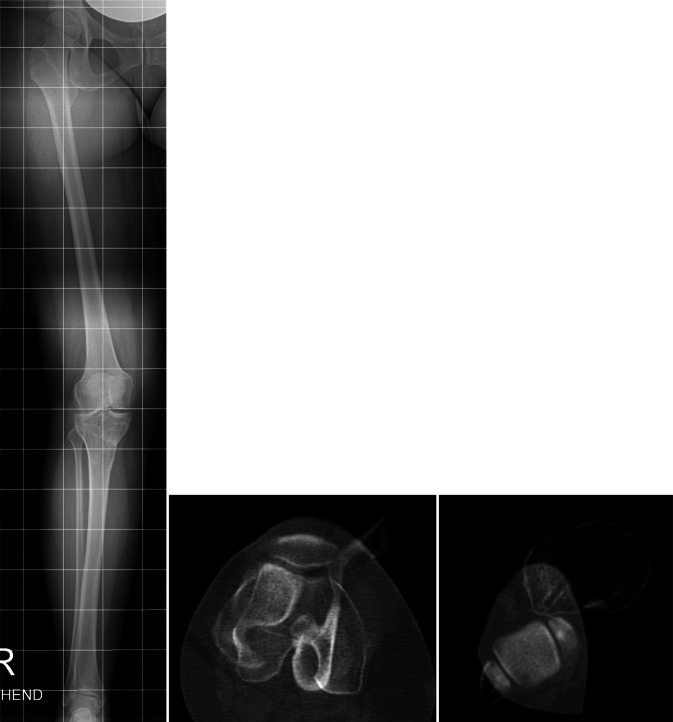
Fall 2: Präoperative Bildgebung, Ganzbeinröntgen sowie schematische Darstellung der Vermessung der Rotationsfehlstellung

35-jährige Patientin mit chronischen patellofemoralen Schmerzen und abnormem Gangbild. Zugrundeliegende Patholgien waren ein iatrogener tibialer Valgus bei Z. n. medialer „open-wedge“ HTO, eine angeborene femorale Maltorsion und eine normale tibiale Torsion (Abb. 7).

Der Behandlungsplan war wie folgt:

1. Femorale Derotation (von 50 auf 25°)
2. Tibiale Varisierung (mcw-HTO, MPTA von 96 auf 87°)
3. Tibiale Innenrotation (von 25 auf 10° AR)

**Abb. 8:**
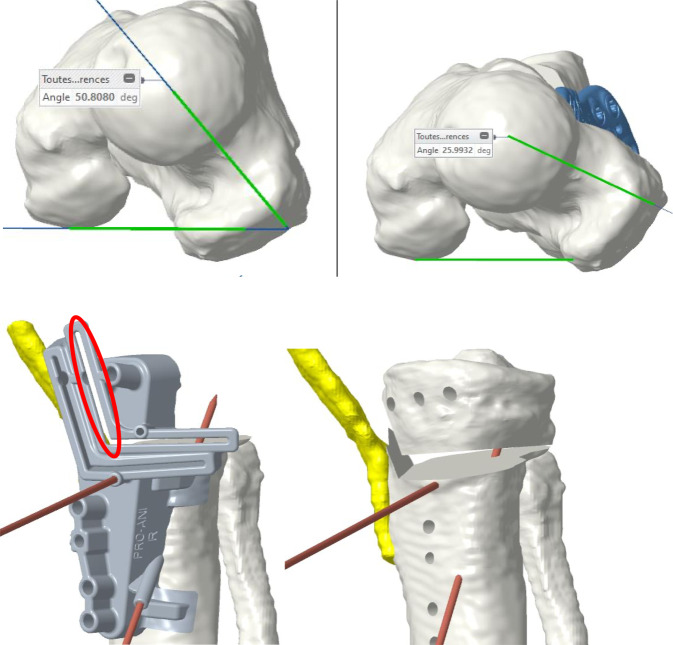
Fall 2: Präoperative 3‑D-Rekonstruktion und Operationsplanung mittels patientenspezifischer Schnittblöcke

Die tibiale Gegenrotation wurde als notwendig empfunden, weil es bei alleiniger femoraler Derotation von 25° zu einem zu drastischen „toeing-out“ gekommen wäre (FPA). Die Umsetzung war mit patientenspezifischen Schnittblöcken vorgesehen (Abb. 8).

**Abb. 9:**
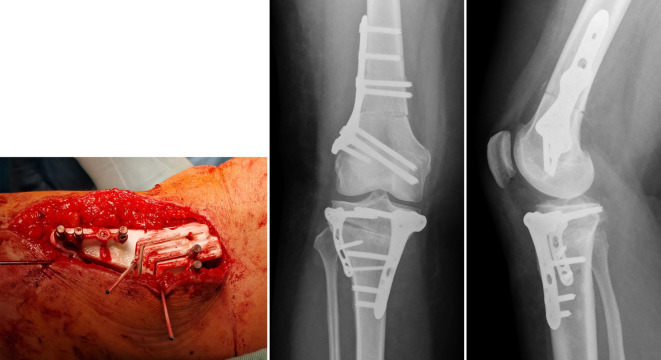
Fall 2: Intraoperativer Situs mit patientenspezifischen 3D Schnittblöcken sowie postoperative Röntgenbilder nach femoraler Derotation und tibialer Varisierung und Derotation, eine zusätzliche laterale Platte zur Stabilisierung des Spaltes lateral

Die intraoperative Umsetzung entsprach weitgehend dem präoperativen Plan. Aufgrund von kleineren Ungenauigkeiten bei den vier tibialen Sägeebenen kam es nach Reduktion der Osteotomie zu einem Klaffen des Spaltes lateral. Daher wurde zusätzlich eine kleine Platte lateralseitig verwendet (Abb. 9).

Die Patientin ist mit dem Ergebnis sehr zufrieden. Das Gangbild ist verbessert und die Versorgung der Gegenseite steht aktuell an.

## Fazit für die Praxis

- Pathologische Torsionen des Ober- und Unterschenkels sind häufig und wirken sich negativ auf das Kniegelenk aus.
- Die physische Verdachtsdiagnose sollte immer mittels Schichtbildgebung verifiziert werden.
- Derotationsosteotomien zeigen vielversprechende Ergebnisse im Sinne von verbesserten „patient-reported outcomes“.
- Welche Operationstechnik überlegen ist, sollte Ziel von zukünftigen wissenschaftlichen Studien sein.
- Patientenspezifische Schnittblöcke haben sich für komplexe Deformitäten, die in mehreren Körperebenen korrigiert werden sollen, bewährt.

## Abkürzungen

FPA: Foot progression angle
TFA: Thigh foot angle
KOOS: Knee Injury and Osteoarthritis Outcome Score
VAS: Visuelle Analogskala
KSS: Knee Society Score
WOMAC: Western Ontario and McMaster Universities Osteoarthritis Index
SF-12: Short Form 12
TT-PCL: Tibial tuberosity to posterior cruciate ligament distance
TT-TG: Tibial Tuberosity-Trochlear Groove distance
